# Facial Skin Temperature and Discomfort When Wearing Protective Face Masks: Thermal Infrared Imaging Evaluation and Hands Moving the Mask

**DOI:** 10.3390/ijerph17134624

**Published:** 2020-06-27

**Authors:** Antonio Scarano, Francesco Inchingolo, Felice Lorusso

**Affiliations:** 1 Department of Medical, Oral and Biotechnological Sciences, University of Chieti-Pescara, 66100 Chieti, Italy; felice.lorusso@unich.it; 2 Department of Interdisciplinary Medicine, University of Bari “Aldo Moro”, 70121 Bari, Italy; Francesco.inchingolo@uniba.it

**Keywords:** N95 respirators, surgical mask, protective face masks, COVID-19, Infection control, Infrared Imaging

## Abstract

Individual respiratory protective devices and face masks represent critical tools in protecting health care workers in hospitals and clinics, and play a central role in decreasing the spread of the high-risk pandemic infection of 2019, coronavirus disease (COVID-19). The aim of the present study was to compare the facial skin temperature and the heat flow when wearing medical surgical masks to the same factors when wearing N95 respirators. A total of 20 subjects were recruited and during the evaluation, each subject was invited to wear a surgical mask or respirator for 1 h. The next day in the morning at the same hour, the same subject wore a N95 mask for 1 h with the same protocol. Infrared thermal evaluation was performed to measure the facial temperature of the perioral region and the perception ratings related to the humidity, heat, breathing difficulty, and discomfort were recorded. A significant difference in heat flow and perioral region temperature was recorded between the surgical mask and the N95 respirator (*p* < 0.05). A statistically significant difference in humidity, heat, breathing difficulty, and discomfort was present between the groups. The study results suggest that N95 respirators are able to induce an increased facial skin temperature, greater discomfort and lower wearing adherence when compared to the medical surgical masks.

## 1. Introduction

Coronavirus disease (COVID-19) is an infectious mild to moderate respiratory illness caused by a newly discovered coronavirus [1,2]. This infection is a serious disease in patients with other pathologies, especially in older people with underlying medical problems such as chronic respiratory disease, cancer, cardiovascular disease, and diabetes. These patients develop serious acute pneumonia with a high mortality rate [3]. A primary way in which the virus spreads is through droplets of saliva, produced during coughs or sneezes or through discharge from the nose from an infected person. Airborne bacteria or viruses can spread infectious diseases, which can become major public health concerns. In particular, tuberculosis (TB) and influenza are major problems in clinical practice [4]. These diseases can be a hazard which can infect care workers. For this reason, it is important to implement airborne infection control by using a good prevention strategy in health-care sites. In fact, the exhaled air of infected humans is one of the prime sources of ambient contamination by bacteria or contagious viruses. Droplets are also particularly dangerous for possible transmission when there is a virus, such as influenza, in high concentrations of airborne particles in closed or small environments [5]. In case of a pandemic involving an airborne-transmissible agent, doctors must use a mask for protection. It is important to evaluate the flow of air through the respirator to understand if there are any points of concern for the health of the doctors. However, the use of protective face masks (PFMs) will not be effective if masks are not used appropriately. Due to resistance to airflow and discomfort related to buildup of facial heat, especially in hot and humid weather, many people use a PFM with lack of compliance to safety regulations [6,7]. The direct surgical mask has a low/moderate filter performance with lower levels of airflow resistance, while the high heat and humidity under a PFM can cause moisture to condense on the outer surface of the PFM, which consequently impairs respiratory heat loss and imposes an increased heat burden [8]. The factors that reduce the discomfort of heat on the face are nasal breathing, use of exhalation valves, reduction of PFM dead space parameters, and cup-shaped or duckbill designs. It has been suggested that facial temperature augmentation can trigger a panic disorder caused by elevated CO_2_ levels under the PFM, with hot flashes and sweating. In fact, wearing a surgical mask or respirator produces a significant increase in skin temperature, especially under the mask. For some subjects in a workplace, this could be sufficient to cause thermal discomfort. A PFM induces a significant augmentation of facial skin effects on thermoregulation. For this reason, many people use an PFM incorrectly, without covering the nose, or, after a few minutes, this can lead to partial uncovering of the nasal area. Impatience with the thermal effects of PFM leads to discomfort and can induce a decreased use and concomitant decreased protection for the user. The purpose of the present study was to evaluate facial skin temperature, discomfort and hands moving the mask when wearing surgical masks or N95 respirators, with thermal Infrared Imaging.

## 2. Materials and Methods

During the study period, February 2020, 20 voluntary male workers met the inclusion criteria with a mean age of 50 (45–55) in the Department of Oral Surgery of the University of Chieti-Pescara, Italy. The study was conducted in observance of the Helsinki Declaration (revised version of Tokyo in 2004) and Good Clinical Practice Guidelines. All patients gave informed consent to the adopted noninvasive procedure. The inclusion criteria were experience in using respirators and absence of respiratory diseases. The volunteers were all in phototypes II or III of the Fitzpatrick scale [9] for the facial areas being evaluated. The exclusion criteria were allergic rhinitis and nasal septum deviations, showing facial aging, lax skin, facial treatment antiaging, severe illness, facial skin disease, head and neck radiation therapy, chemotherapy, facial skin resurfacing, and uncontrolled diabetes. All volunteers had all previously experienced using respirators. In the previous hours, they had not undergone athletic training. 

After a thorough preliminary examination, the volunteers underwent facial temperature evaluation, having been extensively informed about the study procedures. The volunteers were requested to enter a room with a constant temperature for 1 h before the study to allow them to acclimatize. This study was undertaken to investigate the effects of wearing a PFM on facial skin temperature when the subject was not actively working. The perception ratings related to the humidity, heat, breathing difficulty, and overall discomfort of the enrolled subjects were recorded. Discomfort was scored by means of a 100-mm scale from 0 (no discomfort) to 100 (worst discomfort imaginable).

### 2.1. Temperature Measurements

The infrared thermography evaluation was performed in a climate-controlled environment (temperature: 22–24 °C, relative humidity percentage: 50 ± 5%, without any direct ventilation into the mouths of the subjects). The environmental humidity was measured by a built-in integrated sensor (Atmo-Tube, San Francisco, CA, USA). The sensor provided a measurement of relative humidity (RH) at regular intervals with a resolution of 0.5% and a humidity range from 0% to 100%. The facial temperature of the perioral region was recorded by a 14-bit digital infrared camera (FLIR SC660 QWIP, Flir Systems, Danderyd, Sweden). The general acquisition parameters were set with the following specifications: 320 × 240 pixels focal plane array; 8–9 µm spectral range; 0.02 K noise equivalent temperature differences (NETDs); 50-Hz sampling rate; optics: germanium lens; f 20; and f/1.5. The camera was positioned at 0.50 m away from the facial region to obtain the maximum spatial resolution. The thermographic images were recorded at a rate of 10 images per second, and consequently re-aligned by the use of an edge-detection based method implemented with an in-house software package. A thermal video was recorded, and the photos were developed via dedicated software. Temperature changes in the perioral and facial areas were elaborated on the realigned thermal images. The complete act of breathing was recorded by a thermal video, and the temperature changes were calculated by the dedicated software using frame-by-frame records. The average temperature for inhalation and expiration acts were considered for the statistical evaluation. For the thermal evaluations, we considered the emissivity of 0.98 for skin, 0.93 for surgical mask and N95 (any color). The emissivity value is the same for both surgical and N95 because both surfaces are roughened and have the same thermal characteristics. Thermographic data measurements were performed by the software package FLIR QuickReport v.1.2 (FLIR Systems Inc., North Billerica, MA, USA), which is able to obtain the maximum, minimum, and average temperature of a perioral region. 

During the evaluation, the subject was invited to wear a surgical mask or respirator for 1 h and read the newspaper, mainly in silence, speaking aloud for only 10 min. In the first experiment, the volunteer wore a filter type respirator for 1 h. The next day in the morning at the same time of day, the subject wore a N95 mask for 1 h with the same protocol. As a result, there were two variables, no respirator versus respirator, and before and after wearing two different PFM. Skin temperature was recorded before wearing the surgical mask or respirator, during 1 h of wearing, and immediately after having removed the PFM, a video record was taken for another 10 min. Therefore, during protective mask wearing, was a thermal video recorded for 1 h resulted in 10 by 60 by 60 = 36,000 images per investigated subject.

### 2.2. Statistical Analysis

A power analysis was performed using clinical software to determine the number of samples needed to achieve statistical significance for quantitative analysis of facial temperature. A calculation model was adopted for dichotomous variables (yes/no effect) using the incidence effect designed to discern the reasons (85% for the test group and 10% for the control group), with alpha = 0.05 and power = 95%. The optimal number of samples for analysis was 20 patients per group. Numerical results are presented as the ±SD means of all the experiments.

The data outcome was collected and statistically evaluated by the software package Graphpad 6 (Prism, San Diego, CA, USA). The normal distribution of the study data was evaluated by the Shapiro–Wilks test to evaluate the normal distribution. The t-Student test was performed to compare the study variables means in each group. The level of significance was set at *p* < 0.05.

## 3. Results

The videos were converted to infrared images of the facial temperature distribution when wearing the different facemask types. During expiration, the temperature change induced by the airflow appeared in the central area of the mask, while no temperature changes were detected laterally, at the top, or at the bottom of the mask. The superficial area of the surgical mask showed a homogeneous distribution of the heat flow detected by IR during breathing. The N95 respirator group detected a non-homogeneous flow on the mask. The IR images of facial skin temperature distributions were taken during wearing of the mask, immediately after removal of the mask, and 10 min after removal of the mask.

**Figure 1:**
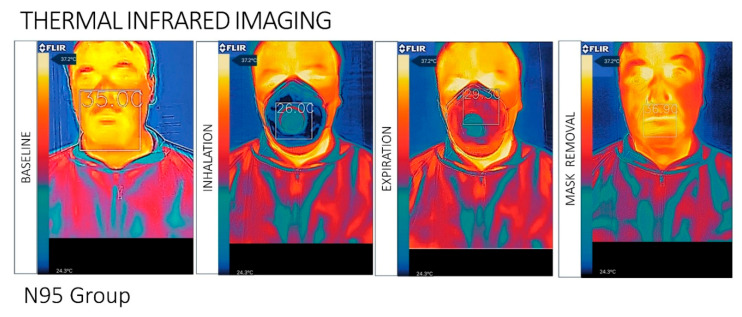
N95 mask group: The infrared images of facial skin temperature distributions associated with the use of a ventilation mask in male subject prior to applying the mask, with the mask applied, and after removal of the mask. The values specified on the thermal images are the local temperatures at the sites of interest (bridge of the nose, two cheeks, and chin), averaged in the respective marked regions (bounded by ellipses). All temperature values are in Celsius.

**Figure 2:**
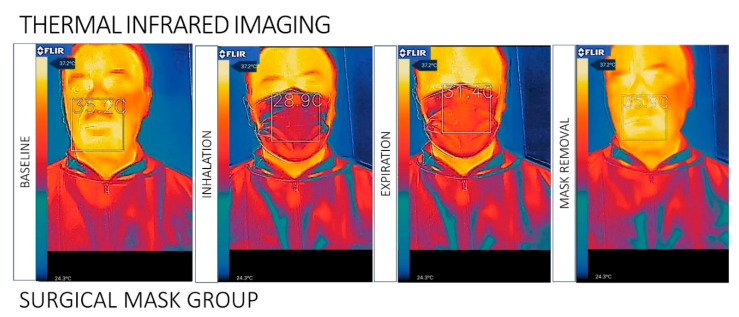
Surgical mask group: The infrared images of facial skin temperature distributions associated with the use of a ventilation mask in male subject prior to applying the mask, with the mask applied, and after removal of the mask. The values specified on the thermal images are the local temperatures at the sites of interest (bridge of the nose, two cheeks, and chin), averaged in the respective marked regions (bounded by ellipses). All temperature values are in Celsius.

The IR thermography images demonstrated significant temperature changes at the perioral region and superior lip immediately after removal of the mask, compared with baseline conditions in both types of PFM. No statistical differences were detected in other regions of the face. Differences were detected in the mask–skin contact sites after removal of the mask, compared with baseline conditions (Figure 1 and Figure 2).

The temperature of the upper lip recovered almost to baseline readings approximately 10 min after mask removal. No temperature augmentations were observed in the forehead, cheeks, and nose/mouth regions. The surgical mask surface showed large temperature changes during inhalation and exhalation (T inhalation: 28.9 ± 3.1 °C; T exhalation: 31.4 ± 3.6 °C).

The N95 respirator surface showed significantly fewer temperature fluctuations during the breathing acts (T inhalation: 26.0 ± 3.6 °C; T exhalation: 29.3 ± 3.8 °C). After the protection device removal, a significant difference in perioral facial temperature was detected (*p* < 0.05), between the surgical mask (mean T removal: 35.9 ± 3.4 °C; ΔT: 0.7 ± 0.5 °C) and the N95 (mean T removal: 36.9 ± 4.2 °C; ΔT: 1.2 ± 0.5 °C) (Table 1 and Table 2).

A statistical difference in discomfort was observed (*p* < 0.01). Additionally, statistical differences were observed regarding the number of touches to the facial mask or face during the 1 h (*p* < 0.05).

Subjects wearing the N95 touched it 25 times to move it, while those wearing the surgical mask performed this gesture 8 times. This underscores the discomfort that a facial mask with a major airflow resistance causes (Table 3).

## 4. Discussion

The outcomes of the present study indicate that fitting a surgical mask or respirator during 1 h of continuous wearing led to an increase in facial skin temperature under the face mask, while removing the face mask tended to rapidly decrease it after 1 min, returning to the baseline after 5 min. A face mask prevents transpiration and protects against airborne transmitted bacteria or viruses and it is very important to wear one in a health care situation, especially during a pandemic [10,11].

This may increase skin temperature irrespective of workload. The increases we observed under the mask were between 0.7 ± 3.3 °C and 1.9 ± 3.5 °C in the respirator. These were lower when the volunteers wore surgical masks. For both types (surgical masks and N95 respirators), increased skin temperature was observed at >34.5 °C, a level which may induce slight sensations of thermal discomfort. On this basis, the larger rise in lip temperature seen in these subjects could possibly be a result of increased airflow resistance to both PFMs. The study size of 20 subjects is sufficient for basic technical hypotheses but is insufficient for the evaluation of other multifactorial effects. For example, we only enrolled healthy subjects. Pulmonary, cardiac, and metabolic pathologies could greatly influence the results of this study. In the present study, we used thermal infrared imaging because this technique is extensively used for evaluating the superficial temperature of bone [12], facial skin [13], and oral mucosae [14].

The increased perioral temperature observed in our study could be explained by the fact that wearing a face mask for a certain period of time causes reduction in heat loss from the body by evaporation, conduction, convection, and radiation [15]. A PFM avoids normal transpiration and cooling of the skin, and the space beneath it (dead space) is filled with warm, moist expired air during most of the breathing cycle. Additionally, surgical masks may increase airway resistance, and a statistically significant decrease in the blood O2 saturation level of surgeons has been found; however, these data were not confirmed by this new study [16]. In this study, we evaluated the effect of facial masks used for 1 h; however, in many situations, masks are worn for longer periods of time. Therefore, a greater effect on the general discomfort of the wearer is conceivable.

Another interesting study has demonstrated an increase in oral temperature when someone is wearing a face mask for a sufficient time, and this condition can influence a wrong diagnosis of fever [16]. In this study, the authors discovered that the subjects wearing and not wearing masks had intraoral temperature above 37.5 and 37.3 °C, respectively, and when the N95 mask was worn the intraoral temperature was statistically significantly different than when wearing the surgical mask. The face is extremely important for thermoregulation of the body; it is two to five times more effective at suppressing sweating and thermal discomfort than the cooling effect of a similar dermal area elsewhere on the body. In fact, the face accounts for 20% of the total drive from the skin and has a high concentration of thermoreceptors [17,18,19].

The facial region and head form an area that is a critical structure for cooling, because is the most sensitive to temperature sensation, whereas temperature sensing is poor on the extremities, with the exception of the fingers, and intermediate in other regions [20,21]. In moderate environmental conditions, such as other areas of bare skin, the surface temperature is about 2–4 °C lower than the internal temperature [22], while temperature gradients from the core to the skin in defined regions, such as fingers or toes, are of 7.0 or even 9 °C, which is not uncommon in healthy people. Perioral and nasolabial region skin temperature in an adult can be around 35.3 ± 1.4 or 35.2 ± 1.3 °C [23]. Body temperature is maintained constant through a combination of physiological mechanisms. In the perioral and nasal region, the PFM that covers the mouth and nose impedes the greater cooling impact of facial skin temperature [24]. Moreover, the straps and head harness of a tight-fitting mask can reduce the venous flow from the head. Many studies have reported that PFMs increase the skin temperature of the lips by 1.9 °C after 15 min without any effect on other regions of the face and little effect on core temperature, and this may have a significant impact on the perception of thermal discomfort [25]. The increase in facial skin temperature induced by PFMs has been documented in different studies, and this significantly influences thermal sensations of the whole body, because cutaneous thermal receptor impulses from the face to the central nervous system are more important than from other regions. The face is the most sensitive region, while the lower extremities (i.e., thigh, calf, sole, and toe) are the least, and it has higher sensitivity to warm temperature and could influence maintaining thermal homeostasis. In fact, when the face of a healthy individual was exposed to heating, local sweating on the leg was augmented three times more than when heating was applied on the leg [17,26].

In this study, we found that the surgical mask produces a slight facial skin temperature augmentation, with more comfort during, and thus increased adherence to, correct use. For this reason, it is better to wear a surgical mask correctly than an N95 which, due to the discomfort, causes displacements with the hands and temporary withdrawals of the mask from the face. A high number of removals of respirators from the face was recorded in the present study, and thermal discomfort may contribute to this. This result should be added to the results obtained by investigators who have shown that wearing a N95 surgical mask does not reduce the risk of infection. In fact, N95 respirators vs. surgical masks as worn by outpatient health care personnel showed no significant difference in the incidence of laboratory-confirmed influenza [11]. The effectiveness of medical masks is not inferior to that of N95 respirators and surgical masks provide similar protection to that of N95 respirators, because respiratory viruses are primarily transmitted by large droplets. N95 respirators are structured to filter against inhaling small airborne particles and fit tightly to the face, while surgical masks are structured against big airborne particles with a loose fit to the face with minor resistance to airflow. N95 respirators appeared to have a larger protective effect than surgical masks, but a recent meta-analysis demonstrated that there were insufficient data to established definitively whether N95 respirators are superior to surgical masks in protecting workers against transmissible acute respiratory infections in clinical settings [27]. In fact, the scientific evidence that N95 respirators are superior to surgical masks is sparse, and findings are insufficient within and across studies [28].

In light of the results reported by our research, a surgical mask presents better adherence and it is better to wear one correctly than an N95, as many studies suggest that the major obstacle against respiratory infections is not the type of PFM worn but the rate of adherence, with a range varying from 10% to 84% [6,7,29]. Another important consideration is the high frequency of touching the N95 observed, which increases self-infection of microorganisms. In fact, contaminated hands are a route to disseminating respiratory infections [30]. The wearing of N95 while not working over the course of 1 h has a significant impact on facial skin temperature, discomfort, and hands moving the mask, which compromises safety and suggests that in working conditions, there is an increase in these parameters.

A limitation of the present study was that all subjects were non-working males and that males present a higher skin temperature than females [31], and we did not investigate the difference between the two sexes; however, none of the volunteers were affected by nasal or respiratory diseases. This study was conducted during the Italian lockdown, and in our department, there were only male patients. Another limitation of this study is that all subjects were wearing the filter on day 1 and the N95 mask on day 2, and we did not randomize order across the subjects to reduce systematic errors. We hypothesize that nasal and respiratory diseases or working increase discomfort during wearing and use of the PFM.

## 5. Conclusions

In conclusion, the N95 mask produces a major increase in skin facial temperature with major discomfort, and volunteers have shown greater adherence in the use of the surgical mask rather than N95.

## Figures and Tables

**Table 1:** Summary of the mask touching frequency, humidity, heat, breath difficulty, and discomfort rate between the two groups (mean, SD. Student’s *t*-test).

	Humidity		Heat		Breathing Difficulty		Discomfort		Mask Touching	
Groups	N95	Surgical Mask	N95	Surgical Mask	N95	Surgical Mask	N95	Surgical Mask	N95	Surgical Mask
Average (SD)	50 ± 8.4	45.01 ± 3.4	60.5 ± 4.22	40.4 ± 6.77	60.5 ± 5.7	30.2 ± 6.33	60.3 ± 3.4	41.5 ± 1.5	12 ± 9.6	5.8 ± 9.6
*p*-value	*p* < 0.05		*p* < 0.01		*p* < 0.01		*p* < 0.01		*p* < 0.05	

**Table 2:** Infrared thermal measurements of the perioral region surface at the baseline during the mask wearing and after the device removal (mean, SD. Student’s *t*-test).

	Temperature Baseline		Temperature Inhalation		Temperature Exhalation		Temperature Mask Removal	
Groups	N95	Surgical Mask	N95	Surgical Mask	N95	Surgical Mask	N95	Surgical Mask
Average (SD)	35.0 ± 2.8 °C	35.2 ± 3.1 °C	26.0 ± 3.6 °C	28.9 ± 3.1 °C	29.3° C ± 3.8	31.4° C ± 3.6	36.9 ± 4.2 °C	35.9 ± 3.4 °C
*p*-value	*p* > 0.05		*p* < 0.01		*p* < 0.01		*p* < 0.01	

**Table 3:** Infrared thermal measurements of the perioral region surface. Temperature differences between baseline and inhalation, between inhalation and exhalation and between baseline and mask removal. (mean, SD. Student’s *t*-test).

	Temperature Difference ΔT_[b-in]_		Temperature Difference ΔT_[in-ex]_		Temperature Difference ΔT_[b-rem]_	
Groups	N95	Surgical Mask	N95	Surgical Mask	N95	Surgical Mask
Average (SD)	1.2 ± 0.5 °C	0.7 ± 0.5 °C	3.3 ± 3.6 °C	2.5 ± 3.3 °C	1.9 ± 3.5 °C	0.7 ± 3.3 °C
*p*-value	*p* > 0.05		*p* < 0.01		*p* < 0.01	

ΔT_b-rem_: Temperature difference between baseline and inhalation; ΔT_in-ex_: Temperature difference between inhalation and exhalation; ΔT_in-ex_: Temperature difference between baseline and mask removal.
